# Epidemiology of Pig Tuberculosis in Argentina

**DOI:** 10.3389/fvets.2021.693082

**Published:** 2021-07-26

**Authors:** Soledad Barandiaran, María Jimena Marfil, Guillermo Capobianco, María Sol Pérez Aguirreburualde, Martín José Zumárraga, María Emilia Eirin, María Ximena Cuerda, Marina Winter, Marcela Martínez Vivot, Andres Maximiliano Perez, Luciano Francisco La Sala

**Affiliations:** ^1^Instituto de Investigaciones en Producción Animal (INPA), Consejo Nacional de Investigaciones Científicas y Técnicas-Universidad de Buenos Aires, Buenos Aires, Argentina; ^2^Cátedra de Enfermedades Infecciosas, Facultad de Ciencias Veterinarias, Universidad de Buenos Aires, Buenos Aires, Argentina; ^3^Departamento de Matemática, Instituto de Matemática de Bahía Blanca- Consejo Nacional de Investigaciones Científicas y Técnicas, Universidad Nacional del Sur, Buenos Aires, Argentina; ^4^Center for Animal Health and Food Safety, College of Veterinary Medicine, University of Minnesota, Minnesota City, MN, United States; ^5^Instituto Nacional de Tecnología Agropecuaria (INTA), Instituto de Agrobiotecnología y Biología Molecular (IABIMO), Instituto Nacional de Tecnología Agropecuaria: Consejo Nacional de Investigaciones Científicas y Técnicas, Buenos Aires, Argentina; ^6^Centro de Investigaciones y Transferencia de Río Negro, Universidad Nacional de Río Negro, Bariloche, Argentina; ^7^Instituto de Ciencias Biológicas y Biomédicas del Sur, Universidad Nacional del Sur – Consejo Nacional de Investigaciones Científicas y Técnicas (CONICET), Buenos Aires, Argentina

**Keywords:** bovine tuberculosis, pigs, spoligotyping, surveillance, farm-level epidemiological variables, *Mycobacterium bovis*

## Abstract

Bovine tuberculosis (bTB) is a disease caused mainly by the *Mycobacterium bovis* and that is endemic to livestock populations in most Latin American countries. Traditionally, bTB control programs are costly and targeted to cattle, largely disregarding other species such as swine and wildlife. According to official services, in Argentina disease prevalence in pigs is comparable to that observed in cattle, suggesting the need for efficient control programs to manage the disease in both species. Additionally, extensive farming systems, which are commonly practiced in Argentina, allow the interaction between livestock and wildlife such as wild boar (*Sus scrofa*), which is considered a natural host of the disease. Here, we evaluated the bTB pigs- cattle interface, studying the dynamics of *M. bovis* isolates in the pig population and identifying farm-level epidemiological variables associated with the disease confirmation at slaughterhouses. Additionally, to assess the potential multi-host systems in the transmission of bTB, the molecular characterization of wild boar mycobacterial strains was included in the study, as this interaction has not been previously evaluated in this region. Multivariable logistic regression models were used to assess the association between farm-level epidemiological variables (location, farm size, and co-existence with cattle and goats) and bTB confirmation in pig tuberculosis-like lesions samples. Results showed that when cattle were present, the odds of bTB in pigs decreased 0.3 or 0.6% for every additional sow when cattle were present or absent in the farm, respectively. Pigs shared 60% (18/30) of the genotypes with cattle and wild boar, suggesting transmission at the interface between pigs and cattle and highlighting the potential role of wild boar in bTB maintenance. These results provide novel information about the molecular diversity of *M. bovis* strains in pigs in Argentina and proposes the potential relevance of a multi-host system in the epidemiology of bTB in the region. The statistical models presented here may be used in the design of a low cost, abattoir-based surveillance program for bTB in the pig industry in Argentina, with potential extension to other settings with similar epidemiological conditions.

## Introduction

Bovine tuberculosis (bTB) is a widely spread disease that causes far-reaching economic losses through direct impact on animal health, restrictions to trade, confiscation and destruction of meat, and costs associated with the implementation of control programs (1). *Mycobacterium bovis* (*M. bovis*) is the most prevalent etiological agent causing bTB. In developing countries, bTB is often a neglected disease with reemergence periods in domestic animals, wildlife, and humans, thus representing a public health concern (2, 3). Because there are many potential hosts for *M. bovis* and disease incidence and distribution are wide, the implementation of effective control measures is complex in regions where susceptible livestock and wildlife coexist (4).

Domestic pigs (*Sus scrofa domestica*) are susceptible to different mycobacteria, mainly those species included in the *Mycobacterium tuberculosis* complex (MTC) and in the *Mycobacterium avium* complex (MAC). In countries in which infection is endemic, *M. bovis* is the most frequently reported *Mycobacterium* in pigs (5–7). Conversely, in countries where bTB is not endemic, MAC species become relevant (8, 9).

Due to geographic and climatic conditions, livestock production in Argentina is largely extensive based on a mixture of natural grazing and pastures. bTB is endemic in the country, and infection is believed to be associated with dairy cattle (10). Although cattle are the most affected domestic species and have the highest bTB prevalence, pigs can also be affected by the disease (7). The local pig production has experienced a significant growth in recent years, leading to an increment of both meat consumption and international trade (11). Few (11%) producers concentrate 54% of the sow's stock, contributing with nearly 80% of the national volume of produced pork. Therefore, a large proportion of small pig producers concentrate a minor number of sows under poor biosecurity and limited technification. This type of farms coexists with relatively large, intensive farms with high technology and stricter biosecurity levels. Often, this scenario makes the implementation of disease control extremely difficult (12) (SENASA, 2016).

Previous studies conducted in Argentina have suggested the role of pigs in the transmission of *M. bovis* to cattle, especially in farms where pigs and cattle coexist (7). Moreover, whereas bTB has been detected in wildlife populations in the county, including collared peccary (*Peccari tajacu*), axis deer (*Axis axis*), gray fox (*Lycalopex griseus*), brown rat (*Rattus norvegicus*), opossum (*Didelphis albiventris*), and wild boar (*Sus scrofa*), no studies have been conducted to assess the epidemiological role of these species as reservoirs hosts (13).

Wild boar may play a role in the epidemiology of the disease in Argentina, considering that their population is rapidly growing and expanding geographically, the infection is present in this species, and its distribution overlaps that of major livestock production areas (14). Also, it is worth noting that wild boar has been suggested as responsible for the maintenance and transmission of *M. bovis* to livestock in other countries (15, 16).

Argentina has a bTB control and eradication program in place since 1998, that is only mandatory in dairy cattle, goats, sheep and in animals bred for genetic purposes (SENASA, Res. 128/12). Contrarily, this program does not include swine, where bTB confirmation relies entirely on meat inspection at the abattoir, which arguably leads to an underestimation of *M. bovis* prevalence.

Worldwide, several studies have addressed the association between bTB infection and farm-level epidemiological variables in livestock (17–19), but only a handful of studies have explored transmission in pig farming systems (20, 21). With this background, disease surveillance and control strategies for bTB could benefit from the development and implementation of a predictive tool that estimates, with adequate confidence, the probability that a slaughtered pig with tuberculosis like lesions (TBL) is truly infected, given the presence of certain farm-level epidemiological variables.

With this background, an observational (cross-sectional) analytic study was conducted under the hypotheses that: (1) the interface between pigs and cattle plays an important role in the dynamics of bTB in Argentina, and (2) wild boar may act as key maintenance host for bTB in the region.

The aim of this study was to evaluate the dynamics of mycobacteria in the pig population and to identify farm-level epidemiological variables associated with *M. bovis* confirmation in TBL samples detected during slaughter. Additionally, the diversity and frequency of spoligotypes was investigated to elucidate potential bTB transmission among pigs, cattle, and wild boar at the interface between these species. Results presented here will contribute to the evaluation and design of surveillance programs for bTB in swine of Argentina.

## Materials and Methods

### Study Population

The studied samples were collected from slaughtered pigs originated from 135 farms located in the main productive areas of Argentina, including the provinces of Buenos Aires, Córdoba, Santa Fe, and La Pampa. A few (3%) samples came from other secondary production provinces such as San Luis, Entre Ríos, and Santiago del Estero. Farms of origin were representative of the two major production systems in Argentina; specifically, 77% of the studied farms were small pig producers and had <1,000 pigs, whereas the remaining 23% were intensive pig producers with 1,000–4,000 pigs.

### Sample Collection

Special permits for accessing abattoirs were issued by the official authorities (SENASA, National Service of Agri-Food Health and Quality). Samples were collected at four annual visits to a large abattoir in the province of Buenos Aires during an 8-year period (2010–2017). Lymph node sections (~4 × 4 cm) with TBL were collected from slaughtered pigs (*n* = 191). Between 20 and 29 samples were collected annually at each of the four visits. Tissue samples were stored at −20°C until bacteriological and molecular analyses were conducted. Samples were collected after meat inspection by SENASA and according to national regulations; therefore, no ethical consent approval was required.

### Mycobacterial Detection and Molecular Confirmation

#### Bacteriological Culture

Samples were decontaminated using the Petrof's method and cultured in Löwenstein-Jensen and Stonebrink media at 37°C for 60 days (22). Ziehl-Neelsen staining of colonies was performed for the detection of acid-fast bacilli (AFB). Briefly, a loopful of AFB colonies was suspended in 300 μL of bidestilled water, then heated for 40 min. at 95°C and centrifuged at 12,000 rpm for 10 min. The DNA obtained was stored at −20°C until processed.

#### Molecular Confirmation

A total of 5 μL of the supernatant was used as template for PCR. IS*6110*-PCR (23) and IS*1245*-PCR (24) were performed to detect the MTC and MAC, respectively. Isolates positive to IS*6110-*PCR were subjected to spoligotyping (25).

### Statistical Analysis

#### Epidemiological Information

Each slaughtered pig was traced-back to the farm of origin, via the National Sanitary Registry of Agricultural Producers (RENSPA), and epidemiological data related to each farm were obtained from SENASA's official database. Farm-level epidemiological data included location (geographic coordinates), size (total number of pigs and number of sows), and co-existence with cattle. When multiple samples were taken from the same farm in different dates, only the one collected closest to date of epidemiological data collection were considered for the statistical analysis.

#### Multivariable Analysis

TBL samples characterized and confirmed as *M. bovis* were considered as cases (true positives; TB-TP), whereas *M. bovis*-negative samples were considered as controls. Multivariable logistic regression was used to assess the association between farm-level epidemiological variables and the confirmation of TB-TP. First, the linearity assumption for continuous predictor variables was checked following Hosmer and Lemeshow (26). Following, a model was fitted including farm location (geographical coordinates), number of sows (continuous), co-existence with cattle (categorical: yes/no) and the interaction between the last two variables (number of sows × co-existence with cattle) as independent variables. The confirmed status of each sample (case, control as outlined above) was included as dependent variable. The presence of influential data was detected using the Cook's distance (27) for each observation in the model. Two farms were identified as strongly influential on the model and were removed before refitting the model (*n* = 133).

Model selection was streamlined by generating models including all possible combinations of variables using the R package “MuMIn” (28). Here, our main focus was to generate a set of best models for making predictions given new data, while ascribing the principle of parsimony (29). Therefore, the Akaike information criterion [AIC; (30)] corrected for small sample sizes (AICc) was used for model selection. The lowest AICc value was considered evidence of best model fit, and models with AIC_c_ values within 2 units of distance were also considered as competing models. Akaike weights (*w*_i_AIC_c_) were calculated and interpreted as the probability that a model was the best-fitting given the data and the set of candidate models. The strength of evidence in favor of one model over the other was obtained by dividing their *w*_i_AIC_c_. Within each model, variable importance was assessed by evaluating the increment in AIC value if the single term was dropped.

First-order spatial effects were investigated by including the location of each farm in the final model, as “latitude,” “longitude,” and their interaction term. Second-order spatial effects were assessed using scan statistic (31) in the R package “satscan” (32) and fitting a Bernoulli model, where “cases” were represented by farms with TB-TP and controls were those with TBL samples which could not be confirmed. Potential multicollinearity in the models was evaluated using the variance inflation factor (VIF) for each variable using the R package “car” (33), where VIF values ≥5 indicate potential multicollinearity problems.

The association between TB status and independent variables was assessed by estimation of the odds ratios (OR) and their 95% confidence intervals (95% CI). The OR for the interaction term was calculated and interpreted following Hosmer and Lemeshow (26). The presence of influential data points in the multivariable model was assessed using the Cook's Distance. All analyses were performed using the statistical program R (34) and the mentioned packages.

The multivariable model was evaluated using a leave-one-out cross-validation procedure, which allowed the estimation of model accuracy (the model's ability to correctly predict sample status), sensitivity (proportion of correctly classified TB-TP), and specificity (proportion of correctly classified *M. bovis*-negative samples) (35). In all models, a decision probability boundary was set at 0.5, to classify samples as positive or negative.

### Genotyping and Distribution Among Species

Spoligotyping was performed following Kamerbeek et al. (25) using a commercial kit (Ocimum Biosolutions Company, Hyderabad, India). The scanned images of the films obtained were analyzed by BioNumerics (Version 3.5, Applied Maths, Sint-Martens-Latem, Belgium). The observed patterns were compared with those in national and international databases (INTA-CONICET and VISAVET Health Surveillance Center, available at: www.mbovis.org). The spoligotypes obtained in this study and those previously reported for pigs in Argentina (7) were compared to spoligotypes reported in cattle (36) and wild boar in the country. The logical relationships between spoligotypes reported in each species were analyzed using a Venn diagram in the R package “Venn” (37).

## Results

### Mycobacteria Detection and Molecular Confirmation

Out of 191 TBL samples, 130 (68%) were TB-TP and 61 (32%) were TB-negative (included negative cultures and MAC positive cultures). In the TB-TP samples, 6.92% (9/130) exhibited co-infections with MAC species. Of the 61 negative TBL samples, 52 were culture-negative and nine were MAC culture-positive.

### Statistical Analyses

#### Multivariable Models

**Figure 1:**
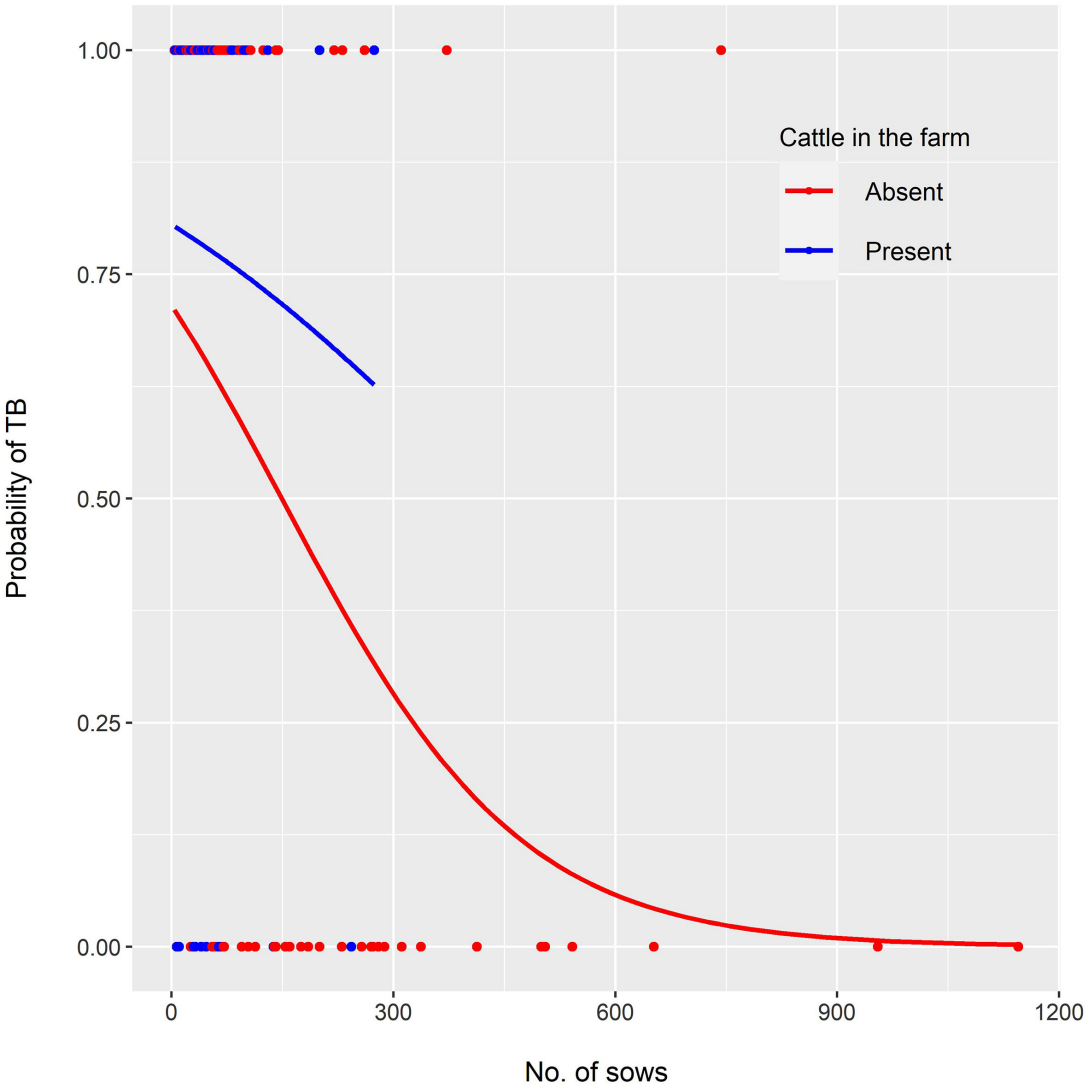
Multivariable logistic model showing the probability of TB confirmation in TBL samples as a function of the number of sows in the farm of origin, when cattle were present or absent (red and blue lines, respectively). Dots along the *x* axis represent samples with (*y* = 1) and without (*y* = 0) TB confirmation, in farms where cattle were present (red) or absent (blue).

Three competing logistic models were selected (Table 1). Their corresponding accuracy, sensitivity and specificity are shown in Table 2. Model 1 showed that the odds of bTB decreased 0.6% (β = −0.006) for every additional sow in the farm but were twice as high (OR: 2.02; 95% CI: 0.88–4.65) in farms with cattle, compared with those without cattle. Model 2 showed that, when only the number of sows was included as independent variable, the odds of bTB dropped 0.7% (β = −0.007) for every additional sow in the farm. Model 3 included an interaction term, indicating that the effect of the number of sows on the odds of bTB depended on the levels of the interacting variable (presence/absence of cattle). In this model, the odds of bTB decreased 0.3% for every unit increase in the number of sows when cattle were present in the farm. When cattle were absent, the trend in the odds of bTB also decreased as the number of sows increased, but the magnitude was 2-fold (0.6%) compared to that observed when cattle was present (Figure 1). No first or second-order spatial effects were detected in the data.

**Table 1:** Parameters of the three candidate logistic models showing the association between risk factors and confirmation of sample TB positive status (*n* = 133).

**Variables**	**Coefficient**	**SE**	**OR (95%CI)**	**ΔAIC_**c**_**
**Model 1** **=** **TB** **~** **No. of sows** **+** **Cattle**
Intercept	0.874	0.339	-	-
Cattle _present_	0.701	0.426	2.02 (0.88–4.65)	0.69
No. of sows	−0.006	0.002	0.994 (0.990–0.998)	11.7
**Model 2** **=** **TB** **~** **No. of Sows**
Intercept	1.234	0.272	-	-
No. of sows	−0.007	0.002	0.993 (0.989–0.997)	18.6
**Model 3** **=** **TB** **~** **No. of Sows** **+** **Cattle** **+** **No. of Sows** **×** **Cattle**
Intercept	0.921	0.357	-	-
Cattle _present_	0.500	0.590	1.65 (0.52–5.24)	1.2
No. of sows	−0.006	0.002	0.994 (0.990–0.998)	9.9
No. of Sows × Cattle _present_	0.003	0.006	0.994 (0.56–1.78)	−1.9

*ΔAICc represents AICc value change when the single variable is removed from the model*.

**Table 2:** Logistic models with their accuracy, sensitivity, and specificity reported as percentage and their 95% confidence interval in parenthesis.

**Model**	**Accuracy %**	**Se %**	**Sp%**	**AICc**	**ΔAICc**	***w*_**i**_AICc**
M1: TB ~ No. of sows + Cattle _(presence)_	72.2	92.7	39.2	159.8	0.0	0.477
M2: TB ~ No. of sows	69.2	92.6	33.3	160.5	0.69	0.338
M3: TB ~ No. of sows + Cattle _(presence)_ +No. of sows × Cattle _(presence)_	71.4	91.5	39.2	161.7	1.89	0.186

*AICc values, differences (ΔAICc), and AICc weights (wAICc) are presented*.

### Spoligotype Diversity, Isolation Frequency, and Hosts

**Figure 2:**
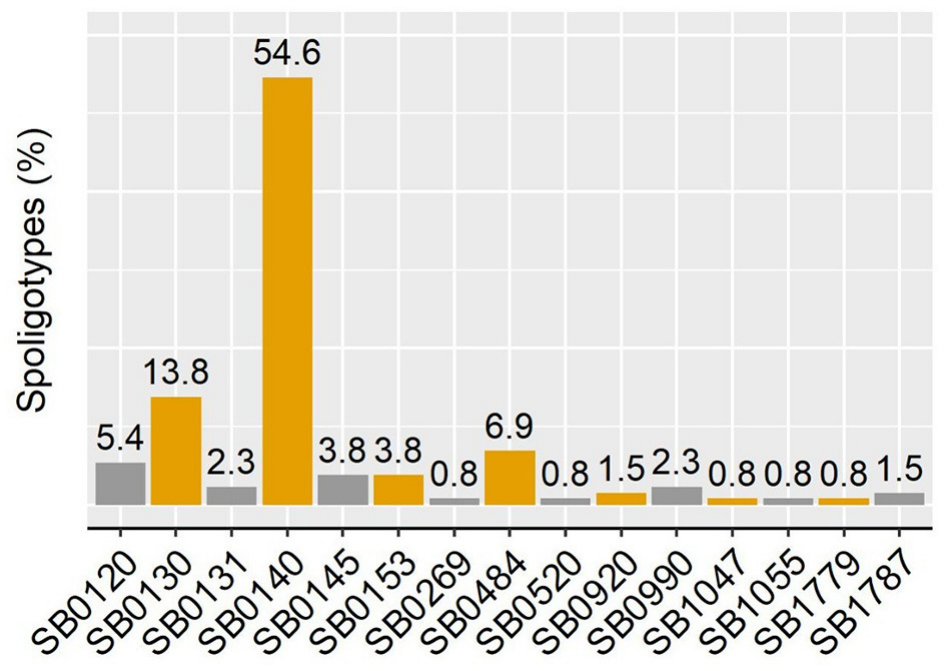
Spoligotype diversity and frequency of occurrence in pigs from this study.

**Figure 3:**
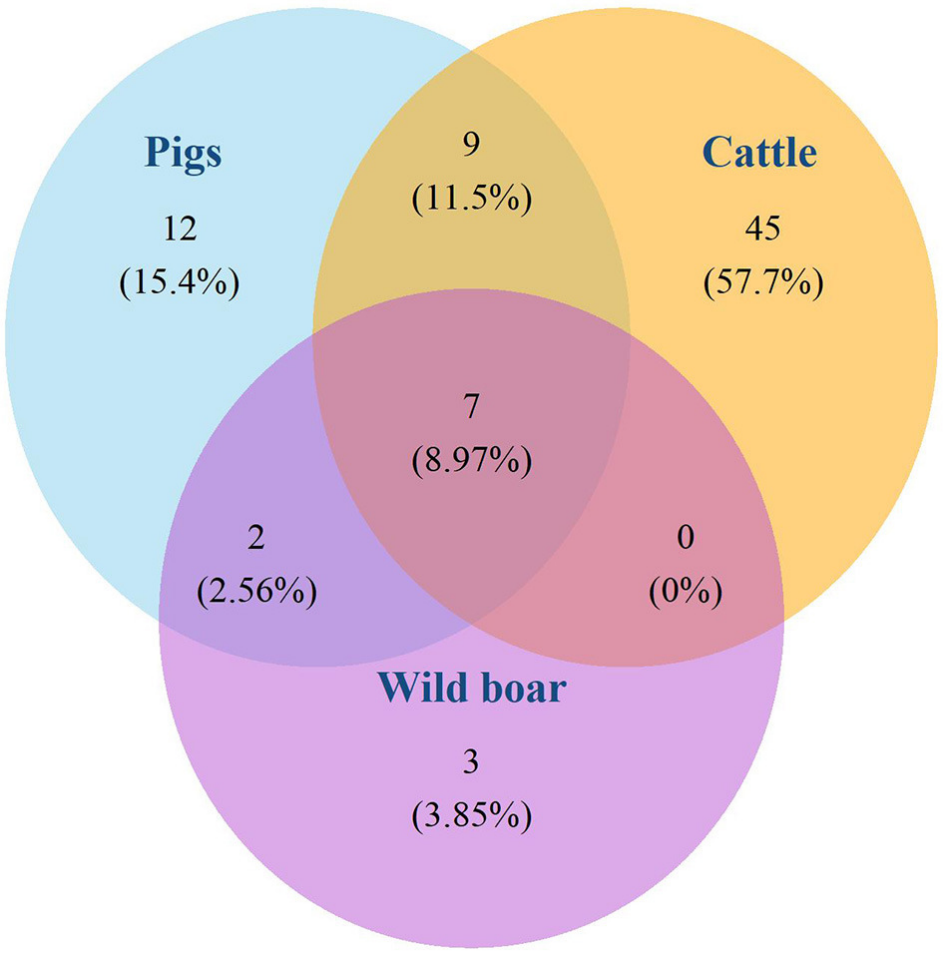
Absolute and relative (%) number of spoligotypes isolated in pigs, wild boars, and cattle, and spoligotypes shared by these species. INTA-CONICET and VISAVET Health Surveillance Center, available at: www.mbovis.org.

The 130 *M. bovis* isolates were classified in 15 different spoligotypes (Figure 2). Unique spoligotypes represented 33.3% (5/15) of the total, whereas 66.6% (10/15) were detected in more than one isolate. The SB0140 was the most frequent (*n* = 71; 54.6%) spoligotype. The distribution of spoligotypes among pigs, wild boar, and cattle is shown in Figure 3. Out of all 78 spoligotypes described here, seven (9%) were shared by the tree species. The frequency of shared spoligotypes between any two species was led by wild boar and pigs (37.5%; 9/24), followed by cattle and pigs (22.7%, 17/75), and cattle and wild boar (10.6%; 7/66).

## Discussion

Despite significant improvement in the last 20 years, bTB and zoonotic TB (zTB) remains a major health challenge for many developing countries. One of the main goals of bTB national control programs is to decrease the incidence of zTB, an objective that can only be achieved through improving food safety and controlling bTB in domestic and wild animal reservoirs (38, 39).

Our study suggests an association between bTB infection in pigs and cattle, through the analyses of the genotypes present in these species and farm-level epidemiological variables. The identification of farms at highest probability of being infected will allow the implementation of effective preventive and control measures in Argentina. Additionally, the diversity of spoligotypes detected in these species strongly suggests the possibility of bTB transmission between livestock and wildlife in this region.

A large proportion (68%) of the collected TBL samples was confirmed as TB-TP. Studies conducted in Spain reported similar results, with over 63% of MTC isolates in TBL samples (40). In South America, epidemiological studies in pigs are scarce, with one study in Brazil reporting that all TBL samples from pigs were negative to MTC isolates (41). Studies conducted in other countries reported lower infection to MTC rates in pigs, such as Ethiopia (10%), Uganda (2%) and Norway (0.3%) (6, 20, 42). Interestingly, Di Marco et al. (43) reported 50% prevalence for MTC isolates in black pigs from Italy and suggested that Sicilian black pigs might act as reservoirs of bTB. The latter seems relevant considering that this breed is produced in extensive farming systems, similar to most pigs in Argentina.

MAC species were isolated in 9.1% (18/191) of TBL samples, similar to previous research in pigs in Argentina (7). In contrast, the percentage of MAC obtained from TBL samples was higher in developed countries compared to the results obtained in the present study (8, 44, 45). Such finding might be related to intensive farming and high biosecurity measures employed in developed countries, where MAC outbreaks are generally associated with contaminated feed or bedding, rather than interaction with domestic animals or wildlife (46). The number of negative samples for both MTC and MAC in this study could be associated with non-infectious causes other non-pathogenic mycobacteria, or different infectious agents (44).

The association between farm-level epidemiological variables and bTB prevalence in pig systems has been assessed in other countries, such as Ethiopia and Norway (20, 42). In our study, the third best model (Model 3) suggests that the odds of bTB confirmation decrease as the number of sows' increases, and that this drop is strongly associated with the absence of cattle. This finding may be explained by stricter health control measures adopted by larger pig producers. Accordingly, in Argentina, small pig producers (50 sows or less) comprise 98% of the registered producers (SENASA 2020). Basic sanitary measures are often neglected by small producers' worldwide (20, 42), and Argentina is not an exception, where only 21% of this stratum is aware of national programs for the control of the most important pig diseases (47). Also, our results highlight the role of cattle in the epidemiology of bTB in pig production systems, and support previous findings suggesting an epidemiological link between coexisting pigs and cattle in the transmission of bTB in Argentina (7).

Genotyping analysis is a valuable tool for the study of bTB dynamics and the role of pigs and wildlife in the maintenance of infection (4, 48, 49). In this study, all MTC isolates were confirmed as *M. bovis*, and 15 different spoligotypes were detected. Here, the most frequent spoligotype was SB0140, which is also the most frequent in Argentina, being detected in zTB and several host species (36).

Our results showed that the frequency of shared spoligotypes was largest between pigs and cattle, followed by those shared between pigs and wild boar. This supports our hypothesis of bTB transmission in a multi-host context, as also previously suggested by others in Argentina and other countries (7, 50–52). Contrarily, the finding of spoligotypes that are not shared with other species (12 in pigs, 45 in cattle, and 3 in wild boar) suggests the relevance of intra-species transmission.

Wild boar shared a large proportion (75%) of its spoligotypes with pigs, suggesting a bTB spillover phenomenon between these species. Wild boar is widely distributed in Argentina, with large populations coexisting in vast areas with pigs and cattle (53). In Argentina, pigs are produced mainly under extensive farming conditions, where cattle presence is also prominent and interaction between wild boar, pigs and cattle can be intense (53, 54). Therefore, wild boar may act as a reservoir in the bTB epidemiology in regions with similar conditions as those in Argentina.

Health research and policy addressing wild boar diseases have received increased attention in recent years (55, 56); however, the role of this species as spreader of bTB and other pathogens is far from being fully acknowledged (54). The surveillance strategy currently in place for bTB in cattle creates a complex scenario where pigs and wild boar continue to play their role as bTB sources for cattle, thus limiting the success of control programs. More in-depth studies are necessary to stablish the role of reservoir hosts in the transmission and maintenance of bTB.

Spoligotyping has limitations regarding its discriminatory power among isolates, and more accurate techniques, such as MIRU-VNTR or Whole Genome Sequencing, would be key to establish infection directionality under the epidemiological context presented here. Also, spatially explicit modeling of different spoligotypes over time might shed additional light on the potential for cross-species transmission of bTB, and specifically on the role of wild boar as reservoir and spillover host. A spatially broader sampling scheme including abattoirs from other geographical regions might reveal spatial patterns in the associations between farm-level epidemiological variables and TB-TP.

In conclusion, the study here suggests an association between presence of cattle and increased odds for bTB in domestic pigs, and also, transmission between domestic pigs and wild boars in Argentina. Results contribute to understanding the epidemiology of bTB in Argentine swine. These results will ultimately contribute to the design and implementation of surveillance and control programs for the disease in Argentina and other settings in which the disease is endemic.

## Data Availability Statement

The raw data supporting the conclusions of this article will be made available by the authors, without undue reservation.

## Author Contributions

SB: data curation, formal analysis, methodology, funding acquisition, writing—review, and editing. MM: data curation, methodology, writing-review, and editing. GC: formal analysis and methodology. MA: data curation and methodology. ME and MV: writing—review and editing. MZ, MC, and MW: methodology. AP: methodology, writing—review, and editing. LS: data curation, formal analysis, writing—review, and editing. All authors contributed to the article and approved the submitted version.

## Conflict of Interest

The authors declare that the research was conducted in the absence of any commercial or financial relationships that could be construed as a potential conflict of interest.

## Publisher's Note

All claims expressed in this article are solely those of the authors and do not necessarily represent those of their affiliated organizations, or those of the publisher, the editors and the reviewers. Any product that may be evaluated in this article, or claim that may be made by its manufacturer, is not guaranteed or endorsed by the publisher.

## Abbreviations

bTB: bovine tuberculosis
TBL: tuberculosis-like lesions
MTC: Mycobacterium tuberculosis complex
MAC: Mycobacterium avium complex
PPD: protein purified derivative test
SENASA, National Service of Agri-Food Health and Quality, AFB: acid-fast bacilli
TB-TP: true positives
AIC: Akaike information criterion
wiAICc: Akaike weight
VIF: inflation factor
OR: odds ratios.
